# Supplementary material for: Determinants and drivers of young children’s diets in Latin America and the Caribbean: Findings from a regional analysis
Source: PLOS Glob Public Health. 2022 Jul 19;2(7):e0000260. doi: 10.1371/journal.pgph.0000260 (PMC10021987; doi:10.1371/journal.pgph.0000260)
Supplement: S1 Text — (DOCX) [file pgph.0000260.s001.docx]

**S1 Text: Literature review search strategy**

| **Search engines** | **Keywords (EN)** | **Publication date** | **Type** | **Relevance criteria** |
| --- | --- | --- | --- | --- |
| 1. Academic databases:  - Google Scholar - Elsevier - LATINDEX - PubMed  1. Organizational websites:  - World Health Organization - UNICEF (incl. global and country-level entities) - World Food Programme - Food and Agriculture Organization of the United Nations - Scaling Up Nutrition Initiative - International Fund for Agricultural Development - Global Alliance for Improved Nutrition - International Food Policy Research Institute | “complementary feeding”, “complementary food”, “child diet”, “child nutrition”, “infant nutrition”, “exclusive breastfeeding”, “formula feeding”, “responsive feeding”, “food system” AND “Latin America”, “Caribbean”, AND *names of individual countries* | 2010 or later | - Peer-reviewed - Gray - Quantitative - Qualitative - Mixed Methods - Original research - Review studies | - Refers to infants/young children - Refers to LAC region - Relevant to one of the objectives of the review |
