# Supplementary material for: Determinants and drivers of young children’s diets in Latin America and the Caribbean: Findings from a regional analysis
Source: PLOS Glob Public Health. 2022 Jul 19;2(7):e0000260. doi: 10.1371/journal.pgph.0000260 (PMC10021987; doi:10.1371/journal.pgph.0000260)
Supplement: S2 Text — (DOCX) [file pgph.0000260.s002.docx]

**S2 Text: Figures with data disaggregated by wealth and residence.**

*Note on all figures: The figures below show the disaggregated prevalence of the indicator for each country using the latest available data since 2010. Abbreviations: ARG: Argentina, BLZ: Belize, BOL: Bolivia, BRB: Barbados, CHL: Chili, COL: Colombia, CRI: Costa Rica, CUB: Cuba, DOM: Dominican Republic, ECU: Ecuador, ELS: El Salvador, GTM: Guatemala, GUY: Guyana, HND: Honduras, HTI: Haiti, JAM: Jamaica, MEX: Mexico, NIC: Nicaragua, PAN: Panama, PER: Peru, PRY: Paraguay, SUR: Suriname, TAT: Trinidad and Tobago, URY: Uruguay. Source: authors’ calculations based on UNICEF Global Database*

**FIGURE 2 DISAGGREGATIONS**

*Fig A. The prevalence of stunting in Latin American and the Caribbean countries, by wealth quintile (lowest vs highest)*

*Fig B. The prevalence of wasting in Latin American and the Caribbean countries, by wealth quintile (lowest vs highest)*

*Fig C. The prevalence of overweight in Latin American and the Caribbean countries, by wealth quintile (lowest vs highest)*

*Fig D. The prevalence of stunting in Latin American and the Caribbean countries, by residence (urban vs rural)*

*Fig E. The prevalence of wasting in Latin American and the Caribbean countries, by residence (urban vs rural)*

*Fig F. The prevalence of overweight in Latin American and the Caribbean countries, by residence (urban vs rural)*

**FIGURE 3 DISAGGREGATIONS**

*Fig G. Exclusive breastfeeding in Latin American and the Caribbean countries, by wealth quintile (lowest vs highest)*

*Fig H. Introduction of solid foods in Latin American and the Caribbean countries, by wealth quintile (lowest vs highest)*

*Fig I. Exclusive breastfeeding in Latin American and the Caribbean countries, by residence (urban vs rural)*

*Fig J. Introduction of solid foods in Latin American and the Caribbean countries, by residence (urban vs rural)*

**FIGURE 4 DISAGGREGATIONS**

*Fig K. Prevalence of minimum meal frequency among children 6-23 months in Latin American and the Caribbean countries, by wealth quintile (lowest vs highest)*

*Fig L. Prevalence of minimum diet diversity among children 6-23 months in Latin American and the Caribbean countries, by wealth quintile (lowest vs highest)*

*Fig M. Prevalence of minimum acceptable diet among children 6-23 months in Latin American and the Caribbean countries, by wealth quintile (lowest vs highest)*

*Fig N. Prevalence of minimum meal frequency among children 6-23 months in Latin American and the Caribbean countries, by residence (urban vs rural)*

*Fig O. Prevalence of minimum diet diversity among children 6-23 months in Latin American and the Caribbean countries, by residence (urban vs rural)*

*Fig P. Prevalence of minimum acceptable diet among children 6-23 months in Latin American and the Caribbean countries, by residence (urban vs rural)*
