# Supplementary material for: Determinants and drivers of young children’s diets in Latin America and the Caribbean: Findings from a regional analysis
Source: PLOS Glob Public Health. 2022 Jul 19;2(7):e0000260. doi: 10.1371/journal.pgph.0000260 (PMC10021987; doi:10.1371/journal.pgph.0000260)
Supplement: Attachment — Submitted filename: LACRO regional analysis PLOS GPH - Response to reviewers.docx [file pgph.0000260.s006.docx]

**Determinants and drivers of young children’s diets in Latin America and the Caribbean: Findings from a regional analysis**

**Manuscript number: PGPH-D-21-00814**

**Response to reviewers**

**Reviewer #1:**

In this article, the authors used a mixed approach to analyze the determinants and drivers of young children’s diets in Latin American and Caribbean (LAC) region. Both desk-based literature review and qualitative interviews with key informants in four LAC countries were conducted. Their findings well summarized the key determinants and drivers for inadequate diets in LAC regions and could be used as cues for program/policy design to tackle the malnutritional problem. However, there are some major and minor concerns that should be addressed before the manuscript can be published.

First, the reason why the four countries (Guatemala, Paraguay, Peru, and Uruguay) were chosen for the key informant interviews needs to be stated in a clearer way. Do they sufficiently represent the dietary situation of young children in the LAC region?

*Response: Indeed, the countries were selected purposefully to include a broad range of dietary and nutrition situations in the region, as well as different socio-economic and policy situations. For example, Guatemala has the highest stunting rates in the region and a high cultural diversity. Paraguay also has a diverse population. Peru is considered a success story in reducing stunting. And while Uruguay is a high-income country; the others are all upper-middle income according to the World Bank classification. We have added the clarification on the country selection in the methods section.*

Second, according to the authors, analysis of key informants interview focused on extracting key emerging themes across six broad topics was performed (Line 133-138). However, in the result (Section 3.2.) only a summary of key informant interview per country was shown. The interview results of each country should be listed in a table and categorized based on each relevant topics, so that readers could compare the results across these countries and identify the common or unique drivers and determinants.

*Response: We have prepared a table with the different drivers of the determinants of children’s diets in the four countries (Table 3).*

Third, in line 44, the authors defined that “undernutrition” includes “stunting, wasting, and micronutrient deficiencies”. However, in line 341 and 343, “undernutrition” and “micronutrient deficiencies” are listed on the same hierarchy. The definition of “undernutrition” should be clearly defined and kept consistent throughout the manuscript.

*Response: Thank you for this observation. We have updated the (old) line 44 to “there is increased risk of undernutrition (stunting, wasting), micronutrient deficiencies and overweight or obesity”, excluding micronutrient deficiencies from the definition of undernutrition. The term is now consistent throughout the manuscript.*

In addition, there are some minor concerns:

1) Figure legends are missing. It is difficult to understand the abbreviations in Fig 2-4.

*Response: Thank you for this observation. We have added figure legends in the revised manuscript*

2) The color contrast for Figure 2 and 4 is not strong. Especially in figure 4, it’s difficult to determine whether the bar charts of “BOL 2016” and “PER 2016” represent “minimum diet diversity” or “minimum acceptable diet”. The color for “minimum diet diversity” or “minimum acceptable diet” is too close to be distinguished.

*Response: Thank you for this comment. We have now included color figures in the revised manuscript.*

3) Line 28-29, the citation of this statement should be included.

*Response: We have added the citation (State of the World’s Children 2019)*

4) Line 62, the full name of “ANC” should be showed when this abbreviation appears at the first time.

*Response: We have written out this abbreviation in the revised manuscript.*

**Reviewer #2:**

Summary and overall impression of the research

This article aimed to identify determinants and drivers of children’s diets in the Latin American and Caribbean (LAC) region with a regional analysis. The authors address an important topic, especially given the current triple burden of malnutrition, current disparities, and budgetary constraints within the LAC region which urge for context-specific understanding and actions. This article holds much promise, and the authors are off to a good start as they employ multiple methods, and make use of a helpful framework to guide the identification of drivers, and structuring of recommended actions. Furthermore, they selected and interviewed key informants of different organisations and levels of influence, of multiple countries which differ in terms of socio-economic and demographic development within the LAC region. The authors share several very interesting and important contextual insights on drivers of children's diet, and actions for addressing the double (or triple) burden of malnutrition in the LAC region.

There are however multiple issues throughout this article which require major revision.

In the introduction, the authors make a strong argument for focussing on disparities within the LAC region, however there is limited and inconsistent continuation of this perspective in the remainder of the article. Most importantly, the formulated research aim does not fully capture all study components. The authors are advised to formulate a list of multiple sub-questions/objectives which represent all utilised study methods. Furthermore, some of the concepts such as determinants/drivers and children’s diet are not well-defined, and used interchangeably which makes it difficult for the reader to extrapolate the key findings and conclusions.

*Response: We have adjusted the paragraph by linking the objective to the study components and added the second objective of the paper: “to identify existing strategic actions that improve children’s diets in the region”.*

*The notion of disparities is already included in the introduction by noting that we “explain why child malnutrition continues to be a problem in some countries but not in others and provides lessons on how to improve the diets of young children in the LAC region”*

*We have added a few sentences on the determinants and drivers to clarify these concepts: “The determinants of young children’s diets during the complementary feeding period include adequate complementary foods, adequate complementary feeding practices, and adequate nutrition services. Access to adequate food includes physical and economic access to nutritious, safe and affordable foods. These determinants are shaped by context-specific factors – referred to as drivers. Together, the determinants and drivers of young children’s diets determine children’s ability to enjoy nutritious, safe, affordable and sustainable diets that protect, promote and support survival, growth and development.” Lines 69-75.*

The methods section is clearly structured, and the analysis approach of key informant data is well-described. However, this section currently does not contain sufficient detail for the author’s study to be accurately replicated by other researchers. Furthermore, this article currently reads as a “multiple methods” study instead of a “mixed-methods” study. There is currently no integration, merging or embedding of the different data sources. Also, a more detailed description on data and methodological triangulation processes would increase the credibility and validity of research findings. Lastly, the process of obtaining ethical consent currently lacks detail.

*Response: We added the following sentence to the methods section: “We integrated findings from the desk review and the quantitative analysis into the country qualitative analysis to triangulate findings from the expert interviews with the available data and country-specific literature.” We also removed the term ‘mixed-methods’ study from the manuscript.*

*We have expanded the section on the ethical consent procedure: “Interviews were completed by three members of the research team, while the analysis was conducted by two other researchers to avoid bias in the interpretation of the interviews. All interviewers had no relation to the key informants prior to the interview. The interviews adhered to ethical standards as set out by the Netherlands Code of Conduct for Research Integrity (19). Participation was voluntary and consensual. Prior to the interview consent was sought and this was reaffirmed at the beginning of the interview (Appendix C). In addition, provided the participant consented, the interviews were audio-recorded to ensure the statements were captured in the correct context, and stored in a safe environment. Furthermore, the data was treated confidentially and anonymously, and hence no direct reference to the key informant is made in this paper.”*

The overall lack detail in the methods section translates into a fairly chaotic presentation of the results and discussion on determinants and drivers of children’s diets. It is unclear which type of literature they focussed on and whether the results of the literature review are exhaustive. Tables which summarise the selected literature would be helpful here to provide context about the presentation of findings, as it is not clear what studies were identified and in relation to which outcomes. Furthermore, the authors should consider only presenting their own findings in the results section. They’re currently referring to other literature in the results section which is preferably positioned in the introduction/discussion section.

*Response: Thank you for this comment. In our view, the literature review was an essential part of our methodological approach, and, hence, part of the data collection process. Therefore, the results of the review are provided under the results section, and not in the introduction or discussion. The goal was to identify literature or previously reported findings on the determinants and drivers of children’s diets in the LAC region.*

*We have further clarified the methods section as per your comments and suggestion below and hope that the revised manuscript is now clearer.*

While the summarised conclusions are succinct, these only address part of the aim and findings, and only refer to common themes. The conclusions on drivers of young children's diets, and implications for future actions also do not represent the disparities between and within countries in the LAC region.

*Response: We have elaborated on the findings that relate to the existing disparities between and within countries.*

Lastly, while this article was generally written in an intelligible fashion and in standard English, it could still benefit from proof-reading before submitting the subsequent version as ambiguous language was used in multiple sections.

*Response: We have carefully reviewed the text and hope to have addressed the reviewer’s concerns.*

Please find below a more detailed list of the major and minor issues for the authors to consider and address in the subsequent version:

Major issues

1. Abstract

1.1. The authors present the scope of the problem, however it would be helpful if they also introduce child feeding which is currently not well-represented in the abstract.

*Response: Thank you, we have added this term in the abstract.*

1.2. Please state in the abstract that you’ve conducted a descriptive analysis.

*Response: We added the term descriptive in the abstract*

1.3. The results and conclusions focus on key themes, however not all of these are common, based on the findings. There is limited information on disparities between and within countries for nutrition, and diet outcomes and related determinants/drivers.

*Response: We have added a statement on disparities between and within countries in the abstract*

1.4. The concepts presented in the key findings would benefit from more detail. For example, from reading the abstract it is unclear what type of “inequality” the authors refer to.

*Response: this has been clarified by our statement on disparities.*

2. Introduction

2.1. The introduction is missing a section which signifies the need for this study. While the authors do a good job of setting the stage, a section which summarises the latest evidence, and how their work is filling a gap would be helpful here. There is also no rationale for the need of a literature review, or this is hidden in your wording. Please make this explicit in your introduction.

*Response: We have added the following sentence: “Against this background, it is imperative to understand determinants and drivers of children’s diets and to identify effective interventions improve the diets of young children. This is particularly relevant since the progress against the key nutritional Sustainable Development Goals (Target 2.1 on ending hunger and Target 2.2 on eliminating malnutrition) has been slow (8).”*

2.2. It is currently unclear what the authors mean with “determinants” and “drivers”. Please clarify the difference in the introduction. This terminology is also not consistently used in the remainder of the article.

*Response: We have added the following clarifications in the introduction:*

*“The determinants of young children’s diets during the complementary feeding period include adequate complementary foods, adequate complementary feeding practices, and adequate nutrition services. Access to adequate food includes physical and economic access to nutritious, safe and affordable foods. These determinants are shaped by context-specific factors – referred to as drivers. Together, the determinants and drivers of young children’s diets determine children’s ability to enjoy nutritious, safe, affordable and sustainable diets that protect, promote and support survival, growth and development.”*

2.3. The research aim is not comprehensive in its current state. For example, the aim does not capture the author’s descriptive analysis. Consider formulating a list of multiple sub-questions/objectives which link to the used methods.

*Response: We have re-formulated the paragraph on the aim of the paper, more clearly specifying the different elements of our analysis.*

2.4. Line 38, the authors state they used a mixed-methods approach but do not explicitly state what they mean by this. Based on the analysis and presentation of the findings this is a multiple methods study. There is no merging, integrating or embedding of the different research methods.

*Response: To some extent, the different research methods complement each other, rather than can be integrated. For the country case studies, there have been more efforts to integrate quantitative data, literature from the review and results from the qualitative interviews. We have revised the text to refer to ‘using multiple research methods’.*

3. Methods

Descriptive analysis

3.1. It is currently unclear where the authors retrieved the malnutrition rates from. The reference directs me to child feeding statistics. Please clarify this.

*Response: we have now clarified which databases were used to extract the malnutrition and complementary feeding indicators.*

3.2. The methods section currently misses a paragraph which provides a justification for the selected data sources for the descriptive analysis. It would be helpful to present an overview of current publicly available data sources on child nutrition and diets.

*Response: see above, we have clarified the source of the data, which are the most prominent sources for these kinds of data.*

3.3. Please specify which software and commands you’ve used to conduct the descriptive analysis.

*Response: We extracted the data and prepared visualizations using Excel. This is now added in the manuscript*

Literature review

3.4. It is currently unclear what type of desk-based literature review is conducted. Please clarify this, and the appropriate supplementary materials including a guideline (e.g. PRISMA).

*Response: We have clarified that our review was a narrative review.*

3.5. Please list all visited databases and used search terms so that others will be able to replicate this review. It would also be helpful to supplement a table which includes the applied search strategy. A table which includes the used term translations would also be very helpful for future research.

*Response: We have further clarified the protocol for the search in the manuscript and included a Table as supplementary material which lists the databases, and search terms.*

3.6. Please clarify which types of academic literature you included (e.g., quantitative, qualitative, mixed-methods)

*Response: We have clarified in the revised manuscript that we imposed no restrictions on the type of articles. We have also included the search protocol as Appendix A.*

Key informant interviews

3.7. Please provide more detail on the interviewing process. Will there be any guides attached for the reader to access? It would be valuable to see how the guides differed based on the stakeholder and supplement these with the article.

*Response: the guides will be included as supplemental material (Appendix C). Note that due to the semi-structured nature of the interviews, we used a single main guide for all interviewees, and interviewers were allowed to deviate or skip parts based on the expertise of the informant.*

3.8. The description of the key informant interviews requires more detail. Currently, a section is missing on the recruitment process of key informants, and the taken quality measures. What did the authors do to enhance the trustworthiness of the findings (e.g., member checking, triangulation, reflexivity). Furthermore who translated the transcripts, and were any cross-translations conducted?

*Response: we have added the requested information to the manuscript: “Interviews were completed by three members of the research team, while the analysis was conducted by two other researchers to avoid bias in the interpretation of the interviews. All interviewers had no relation to the key informants prior to the interview. […] The interviewers, who were also fluent in English, took detailed notes during the interview and then translated the notes into English for the analysis.”*

*The informants’ expertise was ensured by relying on the professional network of local UNICEF offices.*

3.9. The authors mentioned that the analysis focussed on extracting key emerging themes across six broad topics. It would be helpful for the reader when they specify the analysis technique and include the relevant literature (e.g., thematic analysis with a framework?).

*Response: Our analysis most closely followed the deductive content analysis approach as described by Elo and Kyngas (2008). We have added this in the manuscript.*

3.10. The reporting of key informant interviews should be accompanied with a Consolidated criteria for reporting qualitative research (COREQ) checklist or Standards for reporting qualitative research (SRQR) checklist.

*Response: We have checked our manuscript against the SRQR checklist and after a couple of additions, meet the requirements. The checklist is added as a supplementary file (Appendix D).*

4. Results

Descriptive statistics

4.1. I advise the authors to edit the figures by including footnotes which spell out the country names, and insert the appropriate legend labels. The statistics provided in the figures could benefit from stratification by different residential types, socio-economic status of households, and other demographic characteristics if possible.

*Response: Thank you for this suggestion. We have added notes to the figures and now provide figures disaggregated by wealth (lowest vs highest quintile) and residence (rural vs urban) in the Appendix B.*

Literature review

4.2. While this the literature review section focuses on drivers/determinants of children’s diet, the authors often refer to children’s (sometimes not mentioning this either) nutrition status which is not proposed as a study outcome in the research aim. The focus of this section should align with the proposed research aim which is to identified determinants of children’s diets as an outcome of determinants/drivers according to the UNICEF framework.

*Response: Thank you for pointing this out. It was always one of the aims of our analysis. We have now revised our research aim in the introduction and explicitly include nutritional status.*

4.3. Please present the type of methods of your selected studies in appropriate tables so that the reader can assess the strength of evidence on the determinants of children’s diets. It would also be helpful if the selected tables are stratified by country and country groups based on socio-economic development (criteria used for the selection of countries for the key informant interviews)

*Response: Thank you for this suggestion. Since our review was not a systematic review, and only part of a ‘multiple methods study’, we considered it sufficient to provide the results of our review as described in the text, while interested readers could refer to the citations in case they want to look up the cited study in more detail.*

4.4. It would greatly strengthen your analysis when you conduct a cross-comparison analysis on the different countries to bring out the disparities on drivers and actions.

*Response: we have added Table 3 in the revised manuscript, which highlights the key findings on drivers and determinants from the interviews and allows for cross-country comparison.*

4.5. Information on prevalence rates, and other statistics should either be positioned in the introduction (or discussion) to provide context, unless the identified studies presented evidence on increasing rates (for example infant/child milk-based formulas) as key determinants of children’s diets.

*Response: we are unsure to which prevalence rates the reviewer is referring. The second paragraph of the introduction provides prevalence rates of selected child malnutrition indicators for countries in the region.*

4.6. The authors often make general statements about countries in the region (for example, line 235). It would be very helpful when the authors specify the countries.

*Response: We have added country details to line 235 (of the original manuscript) and reviewed the remainder of the manuscript for similar statements.*

Key informant interviews

4.7. Contextual information for each country should ideally be positioned in the methods section (statistics on child undernutrition) when this is not a direct result of your key informant interview.

*Response: We considered the section on the ‘View of experts’ as a key opportunity to integrate and triangulate data from the different sources and methods we used for this study. In addition, such statistics set the stage for the specific countries and provide context for the comments from the key informants.*

4.8. In several sections it is unclear whether a statement represents the view the a key informant, or whether this a general statement derived from literature or made by the author. Please rephrase so that this is clearer.

*Response: we have revised this section for clarity to indicate which statements were made by key informants. We have also added a table (Table 3) with a more detailed list of results from the interviews, to show different drivers and determinants of children’s diets across countries.*

4.9. Consider including participant's quotes, and highlighting different and contrasting views of the key informants of each country.

*Response: we have now added several illuminating quotes form key informants as further examples to some of the statements in this section.*

4.10. The results and discussion sections which cover the key informant’s perspectives could benefit from more detail if the data allows this. For example, it would be valuable to know why there is currently a lack of inter-institutional collaboration and continuity over time in Government’s actions in [country] (line 308) , and more detail on why more progress was made under the prime minister’s responsibility (line 320) to give a few examples. This could really enrich the findings and discussion.

*Response: we have added informant’s quotes to further highlight these and other findings.*

5. Discussion

5.1. The discussion does not contain a section on the strengths and limitations of this study. Please include this in the subsequent version.

*Response: Thank you for this suggestion. We have now included a paragraph on the study limitations in the discussion section.*

5.2. Line 376 – are these new results? I could not locate these statements in the results. Please double-check the discussion to ensure that no new findings are introduced.

*Response: these results are now included in the new Table 3*

5.3. The discussion in some sections is diverging from child nutrition, it is unclear at times what the outcome of interest is. Children’s diets? Or child nutrition status? Please address this. Furthermore, much of the selected literature is not specified to children within the set age-range. This makes it difficult to allocate any of the identified drivers/determinants specific to that age group.

*Response: Although our main interest is children’s diets, children’s nutritional status is a key outcome that reflects the adequacy of children’s diets. Hence we included child nutritional status in the discussion. We have now better stated this as an additional research aim in the introduction.*

*Fair point on the age group. Some studies/findings generalize to children beyond the age range to which we are interested (6-23 months). For example food marketing and ultra-processed foods. However, since this information still plays a key role in shaping children’s (6-23 months) diets, or when they grow older, we think it is important to include these statements in the discussion section.*

5.4. Some statements related to the key informant views are not supported by the findings. For example, the authors state that healthy food is more difficult to access, and more expensive, this is however not reported for all countries, and phrased differently. It would be helpful if the authors double-check this in their subsequent version. Also, please specify which countries you’re referring to when making these statements. Was this for example also mentioned for Peru? It would strengthen your findings and discussion when you highlight the differences between these countries and interpret what this means for future actions in the region.

*Response: Table 3 now provides this information for the 4 case study countries. Based on this table, all 4 countries reported some form of limited access to certain types of food, either through high costs barriers (e.g. Paraguay and Peru) or limited resources form households (Guatemala and Uruguay).*

5.5. In the discussion some countries are mentioned as examples, but it is not clear what and how the disparities are coming forward. It would greatly strengthen this article when the authors discuss these in more detail and interpret what this means for context-specific drivers and actions.

*Response: we have revised some parts of the discussion to more clearly include disparities between or within countries.*

Minor issues

1. Abstract

1.1. It is unclear in some parts which age group the author’s focus on. It would be helpful for the reader when they’re informed about this when reading the abstract.

*Response: We have added the main age group of interest (6-23 months) in the abstract*

2. Introduction

2.1. Please provide more detail on the introduced concepts. For example, it would be helpful to provide definitions of the malnutrition indicators and children’s diets in the introduction.

*Response: we have added definitions of stunting and wasting. We consider ‘children’s diet’ a non-technical term, which is self-explanatory.*

2.2. Consider providing statistics on all forms of child nutrition, including overweight and obesity. It is well documented that most regions are facing double/triple burdens of malnutrition so my advice is to already introduce this phenomenon in the introduction and refer to the latest Lancet series on double burden of malnutrition for example.

*Response: thank you for this suggestion. We provide detailed statistics on overweight in the results section and now state to regional average in the introduction. We didn’t cite the Lancet series on the double burden, as hardly any LAC countries is considered a ‘double burden’ country according to their definitions (see Popkin et al in that series). Their definition includes ‘a prevalence of wasting of >15%, stunting of >30%’, which is not common in LAC.*

3. Methods

3.1. Line 89 – What is meant by “secondary data analysis”? Please clarify this.

*Response: We did not collect our own quantitative data for this study but rather used publicly available data. Hence ‘secondary data’. We have revised the term ‘secondary data analysis’ with ‘descriptive data analysis’ in the revised manuscript for clarification.*

3.2. Please clarify and describe the used snowballing techniques for identif.

*Response: we have clarified the use of the snowballing technique in the revised manuscript.*

3.3. Did the authors use any BOOLEAN terms for your literature search? Consider stating this.

*Response: We have added a clarification in the revised manuscript. We have provided the search strategy in Appendix A*

4. Results

4.1. The descriptive analysis results miss statistics on obesity. If these are not identifiable it is safer to just state overweight within-text.

*Response: In the manuscript, results section, only statistics about overweight are presented in the figures and in the text. There is no mention of obesity.*

4.2. The identified literature which relates to access of food, appropriate services and adequate practices is currently presented across different levels of influence (e.g., individual, household and macro-environmental levels). Consider making this explicit in your writing to make it easier for the readers to follow the narrative.

*Response: Thank you for this suggestion, we have revised the section accordingly*

4.3. Please provide a definition of “access to adequate food”, does this also include economic and physical dimensions?

*Response: We have added this definition in the introduction, when we introduce the UNICEF framework.*

4.4. Line 173 – Consider avoiding ambiguous words such as “encouraging” and “striking” as this is open to multiple interpretations.

*Response: We have revised the sentences for these two terms.*

4.5. Line 205 – this is a very interesting comparison. Consider however positioning this in the discussion. Since you’re looking at disparities within the LAC region, you could you also tailor this example to the price of a similar plate in a higher-middle income country in the LAC region which then highlights the disparities within the region.

*Response: Unfortunately, Guatemala, El Salvador, Nicaragua and Haiti where the only LAC countries included in this study, so we could not compare them to a higher-middle income country in the same region.*

4.6. Line 215 – Is there any literature available on socio-economic differences within rural and urban areas for animal source food consumption? Please also refer to the literature.

*Response: We have added the citation to this sentence (White et al, 2017), this was missing from the manuscript. Unfortunately, this source does not disaggregate its findings by socio-economic status within rural or urban areas.*

4.7. Some sections in the results would benefit from more fluent transitions. For example, the identified qualitative research (refreshing that you include this type of literature!) on WASH services does not build on the previous paragrap and feels therefore isolated (line 238). Please connect this to the narrative.

*Response: We have added a sentence to this paragraph, connecting it to the previous one.*

4.8. Line 256 – Which policies are being referred to here? Please specify this.

*Response: We have now clarified this statement: ‘nutrition education in schools or physical education as part of the curriculum’*

4.9. Please position the table which includes the characteristics of the key informants in the Results section.

*Response: We have made the requested repositioning.*

4.10. Line 284 – The rise of sugar consumption is probably not the sole reason for adoption of regulation strategies, please consider rephrasing this.

*Response: We have rephrased this sentence.*

5. Discussion

5.1. Line 333 – What is meant by a “landscape analysis”? I advise not introducing new terminology in the discussion. Or introduce this type of analysis in the methods section alongside the appropriate literature.

*Response: We have rephrased to ‘regional analysis’.*

5.2. Lines 338-344 – it is unclear whether this section refers to the descriptive analysis. Please clarify this.

*Response: We have clarified that this paragraph refers to the descriptive analysis*

5.3. New concepts and development trends are introduced, such as the triple burden of malnutrition (line 343) and dietary transition (line 338), consider presenting these in the introduction. These are well-established and backed up by a rich literature database.

*Response: We have now introduced these concepts in the second paragraph of the* *introduction.*

5.4. Line 421 – Adopting an iterative process to programme design could be a key conclusion, however the results do not cover this strongly.

*Response: we have added this to the main conclusion on expanding and intensifying awareness campaigns on feeding practices, as it particularly relates to such programmes.*
