# Supplementary material for: Determinants and drivers of young children’s diets in Latin America and the Caribbean: Findings from a regional analysis
Source: PLOS Glob Public Health. 2022 Jul 19;2(7):e0000260. doi: 10.1371/journal.pgph.0000260 (PMC10021987; doi:10.1371/journal.pgph.0000260)
Supplement: Attachment — Submitted filename: LAC regional analysis PLOS GPH - Response to editor.docx [file pgph.0000260.s007.docx]

**Determinants and drivers of young children’s diets in Latin America and the Caribbean: Findings from a regional analysis**

**Manuscript number: PGPH-D-21-00814R1**

**Response to editor comments**

1. Please include your interview topic guide in the main body or as an appendix (an outline of the broad topics, key questions and probes would be sufficient)

*Response: The full interview guide is provided in Appendix C*

2. We can't locate appendix B and C.

*Response: This is very unfortunate. I was able to download these appendices through the online submission portal. Hopefully they are accessible with this re-submission.*

3. Building on ethics - it’d be helpful if you could clarify what you mean by a “safe environment” on p12 – where did you store the recordings? How does audio-recording ensure that they’re statements are stored in a safe environment. I think we know what you mean but it’d be helpful if you could rephrase this is a bit more clearly.

*Response: Thank you for raising this issue. The original sentence indeed needs clarification. We have rephrased the sentence as follows: ‘These audio recordings were stored in a password protected online server and were only accessible to the interviewers to clarify any statements during the analysis stage.’ (lines 189-191)*

4. Table 3 is very helpful! If you could cross-reference to the table more frequently within the body of text, and minimise the text in the table that would improve readability. Lastly, some of the results presented in the table are not included in the main body of text. It would be good if you could double-check this.

*Response: Thank you for this suggestion. We have shortened the text in Table 3 and kept only the key points. We have integrated most of the text in the main body of the text in section 3.2*

5. Words such as “correlation” and “more likely to” were used in the descriptive results section. Unless you did more advanced statistical analyses, you may reconsider the wording – this could lead to confusion among readers.

*Response: Thank you for this observation. We have revised the text accordingly in section 3. (lines 260-261, 295-297)*
